# Welsh Women's Industrial Fiction 1880–1910

**DOI:** 10.1080/09699082.2016.1268346

**Published:** 2017-10-02

**Authors:** Kirsti Bohata, Alexandra Jones

**Affiliations:** ^a^ Centre for Research into the English Literature and Language of Wales, Swansea University, Swansea, UK

## Abstract

From the beginning of the genre, women writers have made a major contribution to the development of industrial writing. Although prevented from gaining first-hand experience of the coalface, Welsh women writers were amongst the first to try to fictionalize those heavy industries—coal and metal in the south, and slate in the north—which dominated the lives of the majority of the late nineteenth-century Welsh population. Treatment of industrial matter is generally fragmentary in this early women's writing; industrial imagery and metaphor may be used in novels that are not primarily “about” industry at all. Yet from *c.* 1880–1910, Welsh women writers made a significant—and hitherto critically neglected—attempt to make sense in literature of contemporary industrial Wales in powerful and innovative ways. This essay maps their contribution and considers anglophone Welsh women writers' adaptations and innovations of form (particularly romance) as they try to find a way of representing industrial landscapes, communities and the daily realities of industrial labour. It identifies the genesis in women's writing of tropes that would become central to later industrial fiction, including depictions of industrial accident, injury, death and disability. And it explores the representation of social relations (class, gender, ethnicity, sexuality) and conflict on this tumultuous, dangerous new stage.

Women writers have been central to the development of the industrial novel, as with Elizabeth Gaskell's *Mary Barton* (1848) and *North and South* (1855), and George Eliot's *Felix Holt* (1866). But the heavy industry that dominated in Wales—coal, iron, steel and quarrying—is notable for its absence in the early canonical novels by women (and men).^1^ These industries were arguably less accessible to women writers with the necessary education and leisure to write than the factories which formed the basis of much of the early industrial fiction. There is a corresponding neglect in critical studies: in Susan Zlotnick's *Women, Writing and the Industrial Revolution*, mining is mentioned just once, in passing relation to *Felix Holt*, and there is a similar dearth in relation to women writers in earlier studies by Martha Vicinus and Catherine Gallagher.^2^ There are some important early literary exceptions. Fanny Mayne's *Jane Rutherford; or, The Miners' Strike* (1853–54) is set in the 1840s and highlights the reforms of the 1842 Coal Mines Act.^3^ The American novella *Life in the Iron Mills* (1861), by Rebecca Harding Davis, is another example of a fictional engagement with heavy industry, although its innovative dreamlike style did not set the pattern for later novels. Frances Hodgson Burnett included some colliery scenes in *That Lass o’ Lowrie's* (1878), which was written partly in Lancashire dialect. In Wales, women writers began to incorporate industrial topography and workers into their fiction from around 1880.^4^ Frequently, the industrial content was a backdrop, an acknowledgement of the industrial features of the landscape in which the characters move, or the adoption of (sometimes detailed) industrial metaphors. In several novels, however, this landscape becomes an important plot device in its own right. When we do find whole novels, or significant sections of a novel, concerned with representing life in the coalfields or quarries, the genre is romance. While this genre eventually closes down industrial conflict in unsatisfactory (implausible) ways, it is capacious enough to provide some vivid scenes below ground and to represent strikes. As feminist critics have argued, reading beyond the formal boundaries of the genre allows us to recognize the social tensions and exposure of ideologies which are present in romance and melodrama.^5^ The contradictions and dilemmas alluded to in these novels include class, gender, and national and linguistic survival—themes centrally relevant to the industrial south, even if they have been overlooked or marginalized in studies of later anglophone industrial writing. In the hands of Irene Saunderson, romance stretches to “presenting the harsh world of the miners and their resistance to the power of the mine owners in a language, clearly, aggressively, in the voice of the oppressed”.^6^

The industrial fictions discussed here are certainly not *working-class* literature depicting “a working man’s consciousness, his consciousness as a working man”, which Raymond Williams made central to his narrowly gendered description of the “Welsh industrial novel”.^7^ The temptation is to write “not yet”, since in surveys of industrial writing, the romances and awkward generic hybrids which use middle-class characters as a conduit for working-class experience published prior to the First World War tend to be presented as a prehistory, a tortuous groping towards (and inevitably measured against) an authentic working-class realism. As Stephen Knight claims: “These first industrial Welsh fictions cannot position themselves for a full, sympathetic and consistent ethnographic account of the new world of industry in Wales as later writers from South-East Wales were to do”.^8^

Undoubtedly, the generic conventions of romance ultimately draw the narrative away from overt industrial issues, and some ideological elements of the novels may be unpalatable to a modern audience, although the writing itself is not without merit. But our central claim is that this body of fiction, in which middle-class female novelists begin to make sense of their industrial regions—and to map the continuities between rural and industrial Wales—warrants a consideration on its own terms. Rather than reading these novels as lacking (in working-class authenticity) or as an immature phase in the development of an appropriate form (when melodrama and romance are abandoned in favour of realism), we try to consider the recurring concerns across the fiction in a more nuanced way. Thus, we focus on the common motifs and generic conventions of those novels by women writers which take their settings, or incorporate scenes and imagery, from industrial life. These include strikes and class conflict, injury and disaster, disability and caring, as well as recurring tropes such as hymn-singing and Christian faith, and conflict between English and Welsh. Many of these are themes which are taken up in later proletarian novels with which we are more familiar.

Many of the novels are out of print and the authors little known, so a short introduction is appropriate here. The five principal writers discussed are almost all from the upper or middle classes. In terms of wealth and social status, Amy Dillwyn (1845–1935) was probably the most privileged. Her father was a prominent Liberal Member of Parliament who owned a zinc works and had other industrial interests in the Swansea area, and metal extraction appears in her fiction.^9^ Dillwyn's novels, most of which were published in a short burst from 1880–87, focus on unconventional, adventurous young women, but her sympathy for working-class grievances is manifest in several scenes in different novels in which local workers turn on their “superiors”. While the industrial element in her fiction is rarely centre stage, she uses realistic scenes of industrial accidents and injury as plot devices. The less wealthy Anne Beale (1816–1900) lived and worked as a governess in Llandeilo, close to the edge of the anthracite coalfield which extended into Carmarthenshire. Her didactic novel *The Queen o' the May* (1880–81) was published by the Religious Tract Society in their Girl’s Own Bookshelf imprint.^10^ Set on the western edge of the coalfields where farmers and colliers live side by side, her novel includes a dramatic pit explosion seen from the women's perspective. Mallt Williams (1867–1950), who with her sister, Gwenffreda, wrote under the pseudonym “The Dau Wynne”, was born into an affluent family near the industrial centre of Swansea, but their novel *A Maid of Cymru: A Patriotic Romance* (1901) is set in Powys among the limestone quarries which supplied the ironworks to the south. Williams was active in the Young Wales and Pan-Celtic movements, and the novel reflects these concerns. Allen Raine (the pseudonym of Anne Adaliza Puddicome, 1836–1908) was the best known of all these writers in her own time. Late in life, she became a bestselling author. Her novels are set in her native west Wales, and she had little direct experience of industrial regions, but *A Welsh Singer* (1897) and, more importantly, *A Welsh Witch: A Romance of Rough Places* (1902) include scenes set in the industrial south. Irene Saunderson (dates unknown) was the only author who wrote an entire novel set in the coalfields: *A Welsh Heroine: A Romance of Colliery Life* (1910).^11^ Little is known about her life, and that little is mainly gleaned from the preface to her novel in which she set down her qualifications for writing about life in the coalfield. As the wife of a doctor, she had lived in colliery communities for a number of years, and so was the closest of all the writers to her subjects. She was also a Welsh speaker,^12^ which may have given her further access to the communities in which she lived; certainly her novel includes more than the usual smattering of Welsh words. Saunderson, who mentions the late Allen Raine in her preface, was consciously writing in a Welsh female tradition, although in plot and elements of style *A Welsh Heroine* bears more than a passing resemblance to Joseph Keating's *Son of Judith* (1900), a romance by a miner turned journalist. A sixth novelist, mentioned here only briefly, is Mabel Holland Grave (1861–?), who published a utopian novel *Fraternity: A Romance* (1888), which is part set in the north Wales slate quarries. Echoing Dillwyn’s call for a rapprochement between the classes in *The Rebecca Rioter* (1880), Grave's imagined cross-class fraternity is to be nurtured by an explicitly Welsh literature and culture.

With the exception of Saunderson's *A Welsh Heroine*, the industrial experience, imagined from without, remains somewhat marginal in the novels discussed here, either textually (the industrial elements take up a few chapters or odd scenes) or spatially (the action often takes place on the edges of the coalfield—although arguably this border zone is itself a significant location). Yet, taken as a whole, Welsh women's industrial fiction is a substantial and pioneering attempt to “novelise”^13^ the turbulent life which had grown up with such dramatic speed around the heavy industries in Wales—spheres of life which were otherwise accessible only through journalism or government reports: “Journals contain frequent accounts of coal-mine accidents”, writes Anne Beale in the course of dramatizing one such, “but outsiders do not realise what they are”.^14^ And there are attempts to represent working-class life, most often from a female perspective: the struggle to care for relatives, to feed a family during a strike, and the agonizing wait for news from the pit.

What we find in these novels is a commentary on industrial relations from a particular bourgeois perspective. Strikes are viewed with regret if not outright hostility, yet the lot of the workers is portrayed as hard and always dangerous. Class conflict is not always a matter of misguided workers against paternalistic owners, as scenes in which soldiers aim their weapons at an unarmed crowd, or a gang of poachers shoot at a landowner suggest, and there is considerable sympathy amongst some writers for the discontents of the workers. The keynote of these novels, however, is rapprochement and fraternity, often framed in a specifically Welsh national context. Mabel Holland Grave gives the most detailed expression in *Fraternity: A Romance*. Part of the novel is concerned with “violent” and “ignorant” quarry workers in north Wales.^15^ Jane Aaron ably explores the novel's ideological allegiances to “young Wales” and “fraternal Socialism”, in which the “classes [ … ] join the masses”,^16^ but for all its “heady vision” of class unity,^17^ one cannot help sympathizing with the “bitter-faced” quarryman who equates the appeal to abandon class enmity with surrender: “It would be very convenient for the gentlefolks if we were all so high-minded”.^18^

In his discussion of “The Strike Novel in the 1890s”, H. Gustav Klaus suggests that there was an “unwillingness of the major practitioners of the novel to engage with industrial conflict at all. Class collision was, if at all, located elsewhere”.^19^ In the case of Welsh women's writing, rural conditions and concerns are often more important than industrial relations, or rather the relationship between mine owner and colliers, manager and men, seems based on the paternalistic models of upper-class landowner and rural tenants. The working class often appear to be more peasant than proletarian. To some degree this overlap reflects the realities of an economy which included agricultural alongside industrial labour for many individuals, sometimes on a seasonal basis. Moreover, the location of mines and quarries in otherwise rural areas meant that, in life and fiction, moors, mountains and other semi-wild spaces were important presences.

Reflecting the overlay of industrial life on older rural class tensions, poaching is frequently represented as a prelude to or substitute for industrial conflict. While poaching may seem a petty offence and lacking in the kind of organized resistance associated with working-class protest, it is, in fact, closely linked in fiction and history with other, more organized, campaigns. In *Chloe Arguelle* (1881), Amy Dillwyn depicts mass poaching raids in which local working men turn skilled night-time hunters, defying landowners, magistrates, the police and even the local Member of Parliament. The novel describes “thirty or forty men, with their faces blackened, spearing salmon by torchlight quite openly. No one attempted to interfere with them and most of the inhabitants of the place quietly watched [ … ] from the bridge”.^20^ In one incident, the poachers open fire on policemen and the Member of Parliament. Although Dillwyn does not use the term “Rebecca”, she is clearly drawing on the salmon-poaching raids which were at their height in the 1870s—described in contemporary newspaper reports as “the revival and reappearance of the terrifying Rebecca and her daughters”.^21^ The gangs adopted many of the same elements of the earlier tollgate protests: organization, intimidation, disguise and ritual. Historically, these poaching “riots” were focused on the upper Wye, rather than more industrialized regions, but the point here is that poaching could become a recognized and effective form of organized protest.

The simmering threat of violence between workers and their overlords is played out in another poaching scene (which, in turn, provokes a strike) in *A Maid of Cymru.* The (English) quarry manager Garry Thoyts catches two of his workers with nets, ferrets and a sackful of rabbits. Acting on behalf of his cousin, the landowner and quarry owner, he confronts them. They refuse to give up their “pay” for their night's work, and a violent fight and chase ensue in which all three are hurt.^22^ Thoyts—with the aid of a passing gentleman—is ultimately victorious. The men are handed over to the law, spend six weeks in gaol for the poaching and lose their jobs, since Garry Thoyts, though no bigot, “had a country gentleman’s instinct about poaching. It was the unpardonable sin in his eyes” (177). The dispute spills over into the quarry, and the inherent class tensions are brought into sharp relief. Since Thoyts will not “condone poaching” by reinstating the men, “[t]he quarry was stopped. War was declared between manager and men” (178).

The motives behind the strike are based on family loyalty and the workers' belligerence: the poachers have “numerous relatives working at Glascwm” and the men are “only too ready, on the slightest provocation, to break out into open insubordination” (177). Yet the willingness of the poachers' “fellow-workers” to “leave the quarry to a man” (177) is also a show of community solidarity—one which the unsavoury English agent who manages the wider estate hopes to exploit. Determined to rid the valley of as many of the “native” Welsh as possible, the agent plans to bring in blacklegs from England, not so much to break the strike as to displace the Welsh from their workplace and homes. As one orator puts it to the men, who have assembled on the martially named mountainside Carnedd y Milwyr (“Cairn of the Soldiers”):

[ … ] this strike of Glascwm quarrymen is no small and simple matter—a mere dispute and difference between officials and men, which ye can settle or refuse to settle as ye choose. It is an affair that will be far-reaching in its consequences, for it affects our Motherland herself. (182)

Leaving aside the point that industrial action is likely to be considered more than “a mere dispute and difference” by those engaged in the material sacrifice and struggles it entails, the strike is central to the main theme of the novel: national survival. The strikers are treated to a lengthy speech by a local bard, in which the Welsh are constructed in racial and linguistic terms, and the men are reminded that they face the danger of “a colony of [ … ] strange men of Somerset settle[d] in your homes”, since it is the “boasted ambition” of the English agent

[ … ] to depopulate the Three Valleys of the “true red blood” and people them with aliens. [ … ] Make him laugh on the other side of his face by refusing to fall into the trap he has laid for you [ … ] and going back to your work in the quarries to-morrow, like loyal sons of Cymru. For Cymru is little and poor and weak, and she cannot lose her strong true-blood sons [ … ] Ye have a heritage to guard, and as you be true men, see that ye guard it well. (183–84)

For their part, the poachers, in this national twist to the strike, will be “proud to sacrifice themselves for the good of their country” (184), compensated by “y Foneddiges” (184)—the gentlewoman and Welsh patriot of the title. Interestingly, the threatened anglicization or colonization of this corner of Wales extends beyond the immediate area of Dyffryn Crawnant and includes the “Three Valleys” of “Rhymney, Dowlais, and Tredegar”, thus casting the “great industrial centres” (31) as actual or potential colonial outposts and, of course, recording the enormous demographic shifts underway in the region.^23^

The six-month strike central to Saunderson's *A Welsh Heroine* is rather more firmly rooted in the economic concerns of the working-class community it depicts. The basis for the strike is a demand for “a five per cent. rise”, although we are given no further context against which to measure the claim.^24^ The workers undoubtedly suffer from the strike: “To villages like Cwmglo, where the majority of the inhabitants depended entirely upon the wages earned in the pits, the gloom was indescribable, a gloom which deepened into utter darkness after a month or two had elapsed” (81). In contrast, the owners are conspicuous by their absence (their home is used by the officers of the cavalry brought in to prevent rioting). Although the aim may be to present both sides, the language associated with masters and men is telling: the masters are “firm” while the colliers are “obstinate” and about to become “idle”, “preferring to plunge their families into the direst want than compromise” (81). The colliers are objects of pity rather than individuals with agency, with the exception of the heroine. During one “mob” scene, the military corners the crowd with near fatal consequences. Unable to disperse because the soldiers are in the way, the crowd listens in fear and confusion as the Riot Act is read and the soldiers take aim. The “mob” is not simply a band of angry colliers, but includes women and children, and thus the whole of the village is held at gunpoint. The narrative focuses on “the women’s shrieks, and the shrill screams of frightened, half-suffocated children [that] filled the air with melancholy” (159). And as the soldiers “raise their carbines” and take aim, the commanding officer (and romantic hero) is prepared for “wholesale slaughter” of “his fellow-creatures who, in their helplessness, were utterly incapable of self-defence or retreat” (162). Melodrama provides a vehicle for Saunderson to expose the lethal class relations that would shortly culminate in the army being deployed in Tonypandy and Llanelli in 1910 and 1911, and, in at least one instance, opening fire. If the crowd is portrayed patronizingly as “poor, deluded creatures” (162), the soldiers' readiness to deliver “a hail of bullets [ … ] riddling them with their molten fire” (163) is damning. Although the officer is not explicitly condemned for his cold-blooded adherence to duty, the dramatic language speaks for itself: “One more word [ … ] and the holocaust of blood and destruction would have commenced” (163). At the height of this crisis, the crowd is saved by the quick thinking and leadership of the working-class heroine, Morfydd Llewelyn. The strike ultimately ends with a compromise on both sides, yet the agency of the owners rather than the colliers is recognized: “conciliatory measures” are “offered and accepted by masters and men respectively” (266). And, by this time, the strike has been overtaken by the unfolding romance plot, which enacts a rapprochement between the classes.

According to H. Gustav Klaus, during the 1890s an “idealised portrait of the working-class leader” became a feature of “the altered approach of middle-class novelists [ … ] to the theme of industrial conflict. Once a ruthless demagogue or imposter, he now appears as the embodiment of integrity and self-abnegation”.^25^ Male working-class leaders are conspicuous by their absence in these texts. There is the shadowy Twm in *The Queen o’ the May*, one of the early agitators who is conveniently killed, along with his sympathizers, in a mine accident. In *A Maid of Cymru*, the foreman tries to keep the peace and persuade the English manager to respect local practices, but his task is really to mediate in the interests of keeping the quarry functional. Instead, the role of leader is performed by dynamic women with imagination and energy. Morfydd Llewelyn and Tangwystyl Hughes in *A Welsh Heroine* and *A Maid of Cymru* are two very different women, separated by social status and motives. But these determined women use similar techniques to provide direction and leadership to crowds of colliers or quarrymen: the power of Welsh hymns.

If this seems a stereotype too far, we need to recall the social milieu from which these novels emerge. In his book on Nonconformist literature, M. Wynn Thomas describes the “visceral splendour”^26^ of the Welsh hymns which formed such an important part of cultural and religious life:

The great hymns and hymn tunes have an incomparable personal and communal power. [… T]hey [ … ] empowered voiceless individuals and bonded people, sucked in from all parts by the vector of industrialization, together into coherent, structured, and orderly communities. These weren't the work of the professional classes. Hymns and melodies were largely the product of shepherds, tinplate workers, blacksmiths, miners and shopkeepers.^27^

In *A Maid of Cymru*, Tangwystyl gathers the strikers on a mountainside, intent on rousing their nationalism above more petty grievances. After the oration given at her request by the Eisteddfodic bard, she sings “songs of the Great Past—and of the Future Hope—the hymns they had first heard as lullabies from mothers’ lips, and those more solemn strains that would be sung over them when their ears were sealed in the great silence” (185), until the men agree to return to work with a cry of “Cymru dros byth!” (185),^28^ thus averting the possibility of being driven out of the valley and replaced by English workers. In Saunderson's *A Welsh Heroine*, the personal and communal strength to be drawn from hymns is dramatized when Morfydd saves the panic-stricken crowd in “a gesture of inspired leadership” by singing “Mamgu’s favourite hymn” and walking “slowly forward to meet the terrible, levelled carbines” (164). The crowd joins in, “obedient as little children”, until “the hymn broke out with all the fervor of the Celtic ‘Hwyl’” (164) and the crowd marches away, past the armed soldiers—indeed, the soldiers mistake the Welsh hymn for a military “march” (173). For all its melodrama, this scene and the wider religious dimensions of the plot^29^ place Irene Saunderson's industrial romance within that body of anglophone writing identified by M. Wynn Thomas as “the first distinctive Anglo-Welsh [literary] ‘formation’—concerned to construct and develop ‘the Nonconformist nation’ by discursive means”.^30^

The historian Philip Jenkins reinforces the importance in Wales “of the hymn as [an] expression of communal solidarity”, arguing that “the love of choral music” engendered by evangelical Nonconformity would become “a vital distinguishing feature of Welsh life, and by no means only for nonconformists”.^31^ Indeed, the centrality of hymns, which are identified by name or quoted at length (in translation), seems peculiar to Welsh industrial writing. Certainly the power of hymns and the act of singing are recurring motifs in women's industrial fiction. In addition to the examples above, in Allen Raine's *A Welsh Witch*, miners trapped underground use hymns to sustain them as they face starvation and madness: “as his trembling voice grew stronger [ … ] some inward calm awoke within him, some strong faith, some hope revived, and with clear and sonorous voice, he sang”.^32^ In some instances, however, the special passion the Welsh are supposed to hold for music can be taken to ridiculous extremes, as in Anne Beale's *The Queen o' the May*. Much of the plot revolves around the passion for music shared by working folk and gentry alike. In this spirit, the mine manager goes down into the pit to try and counter the growing unrest amongst the miners: “I told them that the master was going to give them musical instruments and a trained instructor in the mines”. He is rebuffed: “’Tis more money, not more music we want”, protest some of the men, who refuse to listen to his offer. Almost at once there is an explosion, which the manager and those interested in the musical instruments survive, while the malcontents who have moved to a different part of the mine are all killed. If the moral message was not plain enough, the heroine asks: “Then it was those who were true to their duty that were saved?”^33^

Thus far, the focus has been on the ways in which industrial scenes and settings have been used in conjunction with other narratives—the nationalism of Mallt Williams or depictions of religious faith or (romanticized) female agency—as well as the way wider class conflicts merge with industrial disputes. In the second half of the essay, we turn to look at themes and tropes in women's pre-war industrial fiction which would eventually reappear in men’s working-class writing of the 1920s and 1930s.

Industrial accidents and mining disasters are stock features of coalfields literature, included from the outset in women's writing. Female perspectives are privileged in Anne Beale's *The Queen o' the May*, which features a powerfully related colliery explosion told from the perspective of the frantic families of the victims and rescuers. The tense scene above ground would be repeated by Allen Raine and Irene Saunderson—the transfixed watchers by turns terrified and excited, the waiting punctuated by the movement of the pit cage, disgorging stretchers or taking in heroic rescuers.

The action goes underground in Raine's *A Welsh Witch*. Three men and a newly employed 12-year-old boy are trapped for several days after a roof fall caused by an explosion. They survive the afterdamp, but face starvation and madness. Stephen Knight suggests that Raine was “drawing on Joseph Keating’s 1900 novel *Son of Judith*”, but there is little to connect the two in style or plot.^34^ Jane Aaron instead identifies “accounts of a disaster at Tynewydd Colliery, Porth, in 1877, when four men and a boy survived entrapment underground for nine days”, as the likely source of Raine's story,^35^ and many early novelists seem to have gleaned some basic information from newspapers, government reports or other non-fiction sources.^36^

If Raine focuses on a realistic account of men trapped below ground, the explosions in Anne Beale and Irene Saunderson provide an opportunity for female protagonists to demonstrate courage and strength. In *The Queen o' the May*, the heroine proves the value of her domestic skills, bearing restorative tea to exhausted miners, nursing the sick and injured, and offering emotional support and news to those unable to get to the pit. In *A Welsh Heroine*, when the men have done all they can, Morfydd goes underground to rescue the manager herself.

Although the drama of the fatal underground disaster remains a vivid literary reminder of the human costs of coal, more recent scholarship has focused on the fate of the maimed survivors of accidents and on the representation of disability in coalfields literature.^37^ With a high incidence of industrial accidents and chronic illness associated with the industrial and domestic environments of the coalfields, disability was the norm rather than the exception. Disability along with injury, disfigurement, sickness and the provision of care are important themes in women's industrial writing in this period. Caregiving could be bound up in the conservative gender ideology espoused in didactic writing, as in *The Queen o' the May*, which conforms to David Mitchell and Sharon Snyder's description of Victorian “instructional tales” which “evidence a penchant for discussing disability in terms of individual responsibility and the need for charity [in this case filial duty] towards the infirm”.^38^ Indeed, the heroine's life, and therefore the plot of the novel, is determined by a series of domestic caring duties—for her aged great-grandfather, her cousin who was burned in a pit explosion, and, finally, her mentally ill father—for which she is duly rewarded with marriage and improved social status. Morfydd, in *A Welsh Heroine*, performs a similar role in caring for her frail Mamgu (“grandmother”), although she also earns money by taking in washing to help provide the nourishing food her aged relative needs.

While women remained the primary caregivers in late nineteenth- and early twentieth-century industrial centres, medical intervention and organized welfare systems were increasingly important—the strike in Joseph Keating's *Son of Judith* is to win the right to increase the weekly medical insurance money taken from the miners' pay. Coalfields were important sites for the development of medical services to deal with both emergencies and the longer-term health needs of disabled miners. Irene Saunderson, no doubt informed by her husband's profession, mentions medical details with some frequency in *A Welsh Heroine*, including “our new ambulance service” (38) and the use of “beef-tea tabloids” (310) to sustain overstrained colliers during a rescue below ground. Even the ongoing debate about pneumoconiosis and the health of colliers' lungs seems to lie behind a detailed description of “the blood coursing” in Morfydd's “veins”, made “vigorous and buoyant” due to the mountain breezes “carrying the oxygen so necessary for the manufacture of good arterial blood” (94). Allen Raine tries to make a similar observation, erroneously claiming that, “fortunately for the colliers” who are covered from head to toe in coal dust, “it [coal dust] is not unwholesome, or their lives would be seriously endangered by the clogging of their skin”.^39^ Clean skin was a significant concern for the Victorians,^40^ but inhalation was the real health issue; “by the 1860s it was accepted that miners suffered from a distinctive respiratory disease caused by inhaling coal dust”, although it would be many decades before “miner’s lung” was properly recognized.^41^

In addition to the incidental medical discourse in *A Welsh Heroine*, two of Saunderson's central characters are “disabled”. One is a sympathetically portrayed itinerant grocer with a wooden leg, the other a malevolent figure whose body represents his character. Named for his deformity, “Jack-y-bandy” (bandy-legged Jack) has “crooked legs, cumbered [ … ] with very large, flat feet” (20) that impede his movement, “a cadaverous face” (20) and “a very large head” (11), which lend him an “ungainly form” (13). Jack-y-bandy is an example of the long-standing connection between “sin and physical aberration”.^42^ His body is an outward manifestation of his “stunted nature” (16): “His soul, like his body, was so cramped and warped, until all semblance of manliness had been lost, and in its stead a monster, abnormal and hideous in cruelty and meanness lived” (13).

The “medicalization” of disability which occurred in the nineteenth and twentieth centuries, where impairments were increasingly viewed as “conditions” to be remedied or “normalized”, rested on a “*dichotomy* between normal and pathological”.^43^ Or, to put it another way, “normality” is the condition to which disabled individuals are required to aspire. Jack-y-bandy is arguably restored to physical “normality” and spiritual health via self-sacrifice. Hitherto a bully and a coward, he heroically rescues colliers trapped in the mine following an explosion, before himself becoming trapped beneath a rockfall. Over two chapters, the rescue and his slow death and absolution are described without a single reference to his physical deficiencies (although he keeps his name almost to the end). In the closing paragraphs of the novel, the dead manager is “warmly cherished” (329) for his “self-sacrificing heroism” (330), while in the grave “a *hero’s body* lies, broken and torn” (329, our emphasis). Arguably, the narrative remedies Jack-y-bandy's physical defects by omission in tandem with his spiritual restoration, but it also allows for a disabled body to be a hero's body.

Saunderson's treatment of the more attractive Jeremiah Jinkins is more subtle. Jinkins has a quasi-paternal role in this novel and, as such, is a double for the unpleasant Jack-y-bandy who, it turns out, is Morfydd's natural father. Jinkins finds laundry work for Morfydd during the strike and gives her the occasional gift of money or food, despite his status as a renowned miser. He is not defined by his disability—unlike Jack-y-bandy, who is named for his crooked legs—and neither his amputation nor his prosthesis symbolizes his character or soul, although his leg does render him a slightly comic figure, from the tap-tapping of his walk to his “affectionate” (101) regard and “customary study of his leg” (103), which are linked to his humorous portrayal as a “philosopher” (76).

Disability critics Mitchell and Snyder have argued that disability requires (and engenders) a narrative.^44^ The circumstances of Jinkins' first amputation are not related. Instead, Jinkins and his prosthesis are at the centre of a comic interlude which is intended to introduce us to the wider community in an essentially ethnographic passage.^45^ After some banter with the local women, who will not buy his produce in the face of an impending strike, Jinkins tries to stop his wayward pony from darting off with his merchandise by sticking his leg, “which happened most fortunately to be wooden” (73), into the spokes of the cartwheel. It shatters, leaving “Mr. Jinkins sitting on the ground, with an expression of the supremest surprise upon his usually phlegmatic countenance” (73). The comedy continues as bystanders ask if Jinkins is in pain and congratulate him on using the wooden leg rather than the other leg; eventually the doctor arrives, only to be dismissed: “It be the carpenter I do want, syr” (76). The self-importance of Jinkins deserves to be punctured, but, in fact, Saunderson's treatment of this incident, and in particular the way in which she describes the prosthesis—at once a part of his body and yet an inanimate and replaceable appendage—reveals a more serious interest in the body and its boundaries. The comedy of the scene depends on the dual nature of the prosthetic leg as body part and prosthesis, both “my leg” and a “limb” (74) and, at the same time, a “ragged stump” of wood (73). The fluid status of the prosthesis—the “remnant of what a short time previously had composed part of his anatomy” (73)—is finally underlined in the rather uncanny afterlife of the leg:

The portion that was snapped off from the wooden leg, reposed that night, wrapped up in an old shawl within the arms of a little child who had found it lying a yard or two away from the accident.

“Bye-bye, dollie,” crooned the child, as she hugged her treasure-trove, and closed her eyes for the night. (80)

The ubiquity of industrial accidents and casual “minor” injuries is also utilized as a plot device in Amy Dillwyn's fiction. Disfigurement and disability in an industrial landscape are important elements of the detective plot of *A Burglary, or Unconscious Influence* (1883). The burglary of the title involves a wealthy heiress being tied to her bed by a man disguised as a “poacher”, who then makes off with her jewels. He is, in fact, a gentleman thief, but his working-class disguise—and, in particular, the fact that he is missing a finger—successfully deflects attention onto a local collier and “notorious” poacher. In the eyes of the squire and local magistrate, a poacher is “capable of any iniquity”, and he believes that he has found his man.^46^ In his defence, the miner's wife points out the ubiquity of maimed colliers: “what with drams slipping, and stones falling, and one thing and another, there be always something wrong at them collieries”.^47^

The industrial landscape in which Dillwyn's characters reside is a potential danger in more than one novel. Several characters meet their end in industrial workings: by falling down a disused quarry (*Jill and Jack*, 1887); being thrown down an abandoned mine and left to perish (*The Rebecca Rioter*, 1880); dying from a blow to the head inflicted by refuse falling from a slag heap (“Nant Olchfa”, serialized 1886–87); or being crushed and burned to death by a white-hot steel ingot (in the same novel). This last is a rare instance of a literary description of this kind of industrial accident, and Dillwyn offers a detailed and gruesomely realistic account of the event. The upper-class villain, disguised as a workman, gets into the path of men moving an ingot at speed to the “great hammer which was to beat it into shape”. They try to stop, but the ingot shoots off the trolley:

There was a sound of a heavy body hurtling through the air, a shriek of sudden pain and terror, a crash, and then Reginald lay writhing and screaming on the ground, with his legs crushed beneath the weight of more than a ton of steel at nearly white heat. Then ensued a ghastly scene, whose recollection haunted those who witnessed it for many a long day afterwards.

The first necessity was to procure long iron bars as levers, wherewith people could prize up the superincumbent mass of burning metal from off him, without approaching nearer to its fiery heat than flesh and blood could endure. […]  [W]hilst they were being hunted for and dragged to the spot, the sufferer had to lie in agony, with crushed limbs, and exposed defenceless to the heat that scorched and blistered him cruelly, and yet did not destroy consciousness.^48^

The scene goes on at some length, detailing the man's protracted agonies. Although Dillwyn, like Keating in *Son of Judith*, relies on a story which focuses on the “quality” rather than the ordinary workers, who were the most likely to be injured in the steel or coal industries, pioneering scenes such as this constitute an important and early fictional encounter with the dangers of heavy industry.

The middle- and upper-class women writers discussed here adopted romance as a flexible means to explore the industrial themes, scenes and settings which were transforming Wales—economically, linguistically, ethnically and politically—in the late nineteenth and early twentieth centuries. Irene Saunderson, who was the most immersed in the “colliery life” she wrote about, went furthest in trying to represent the bilingual, Nonconformist working-class world she inhabited with her husband, a doctor in the coalfields. The romantic alliance between an upper-class English officer and a working-class Welsh girl may seem absurd, detracting from both the astute ethnographic detail of her writing and her feisty working-class heroine. But melodrama also allowed Saunderson to represent the heightened class conflict that would, indeed, explode in a lethal confrontation between army and workers shortly after her novel was published. Other women writers were more concerned with a generalized class conflict, seeing the struggle in the mines as connected to wider inequities shared by rural workers, connecting scenes of industrial unrest with riots and poaching in the border zones where industry and rural life merged. Still others found a framework for transposing nationalist resistance onto working-class solidarity; indeed, what is perhaps most striking is the markedly and self-consciously *Welsh* character of all the texts, which seem indebted to the contemporary Young Wales movement. In writing about colliery life, these writers foregrounded disability and care work alongside the more sensational disasters, industrial action and community cohesion which we tend to associate with later industrial literature. Taken as a whole, then, these early fictional representations of heavy industry in Wales constitute an important body of work that expands what we understand as industrial fiction.

## Notes

1. Raymond Williams, “The Welsh Industrial Novel”, *Who Speaks for Wales: Nation, Culture, Identity*, ed. Daniel Williams (Cardiff: U of Wales P, 2003), pp. 95–111 (99). Though set close to a mining district, *Felix Holt* does not depict colliery life. On the wider contribution of women to early industrial writing, see Joseph A. Kestner, *Protest and Reform: The British Social Narrative by Women, 1827*–*1867* (Madison: U of Wisconsin P, 1985).

2. Susan Zlotnick, *Women, Writing and the Industrial Revolution* (Baltimore: John Hopkins UP, 1998), p. 229; Martha Vicinus, *The Industrial Muse:* *A Study of Nineteenth Century British Working-Class Literature* (London: Croom Helm, 1974); Catherine Gallagher, *The Industrial Reformation* *of English Fiction: Social Discourse and Narrative Form, 1832* **–** *1867* (Chicago: U of Chicago P, 1988). Even Kestner, who discusses Fanny Mayne’s novel (below), fails to index mining or other heavy industries.

3. Mayne’s heroine is a seamstress, but also the daughter of a collier and strike leader. Kestner argues that “Mayne attributes fifties reforms to a period in which they were at best initiated” (177). Mayne was editor of *The True Briton* (in which *Jane Rutherford* was serialized); here she also published articles on industrial topics and claimed to have been underground (180). Other early writing about coal includes a short religious tract, *The Lancashire Collier Girl* (1795), by Hannah More. Fiction by men includes Robert Douglas’s (also known as The Medical Student) “The Miners: A Story of the Old Combination Laws” (1844) and G. Wharton Simpson’s “Colliers and Coal Mining” (1851).

4. There are earlier examples of poems and ballads by women which acknowledge heavy industry in Wales. See, for instance, Jane Aaron’s discussion of Elizabeth Davies of Neath in *Nineteenth-Century Women’s Writing in Wales: Nation, Gender and Identity* (Cardiff: U of Wales P, 2007), p. 190. Male novelists were active at about the same time. A Welsh-language novel, *Arthur Llwyd* (1879) by John Thomas, depicts the opening of a coal mine, while Joseph Keating, a former miner, published *Son of Judith: A Tale of the Welsh Mining Valleys* in 1900. In addition, several Wales-set mining novels were published, including Israel Mort, overman: a story of the mine (1873-76) by the Devon-born writer John Saunders and Rhoda Roberts: a Welsh mining story (1895) by Harry Lindsay who spent at least part of his teaching career in Wales.

5. Rachel Blau DuPlessis, *Writing beyond the Ending* (Bloomington: Indiana UP, 1985), p. 19. In their studies of “race melodrama” and “border romance”, Susan Gillman and Rita Keresztesi tease out the ways in which “this genre [romance] accommodates the intercultural, interracial, international, and historically determined personal encounters that make up what Fredric Jameson calls the ‘political unconscious’ of a nation”. Rita Keresztesi, “Romancing the Borderlands: Josephina Niggli’s *Mexican Village*”, *Doubled Plots: Romance and History*, ed. Susan Strehle and Mary Paniccia Carden (Jackson: UP of Mississippi, 2003), pp. 107**–**26 (113). See also Susan Gillman, “The Mulatto, Tragic or Triumphant? The Nineteenth-Century American Race Melodrama”, *Culture of Sentiment: Race, Gender and Sentimentality in 19th-Century America*, ed. Shirley Samuels (New York: Oxford UP, 1992), pp. 221**–**43.

6. Stephen Knight, *A Hundred Years of Fiction: Writing Wales in English* (Cardiff: U Wales P, 2004), p. 30.

7. Williams, p. 102.

8. Knight, p. 31.

9. David Painting, *Amy Dillwyn* (Cardiff: U of Wales P, 1987).

10. The story was first serialized in *The Girl’s Own Paper*, 9 October 1880–9 May 1881 (and a sequel, entitled “May Goldworthy”, appeared there in 1881–82). The single-volume edition was published by the Religious Tract Society in 1881.

11. The date of publication is usually given as 1911, but there is documentary evidence to support the earlier date of 1910. The preface is dated April 1910, and reviews of the novel appeared in the summer of 1910, including one in June in the *Rhondda Leader*, followed by another on 11 August in the *South Wales Daily Post*, while the same paper reported on 30 June that a copy of the novel had been accepted by the King. The earlier date would contradict Knight’s assertion that Saunderson’s riot scene is based on the Tonypandy riots of November 1910–11.

12. “Mumbles Lady in Canada”, *The Cambrian*, 1 Sept. 1905, *Welsh Newspapers Online*, <http://newspapers.library.wales/>.

13. The term is used by Lewis Jones in his preface to *Cwmardy* (London: Lawrence and Wishart, 1937), n. pag.

14. Anne Beale, *The Queen o’ the May* (London: Religious Tract Soc., 1881), p. 131.

15. Mabel Holland Grave, *Fraternity: A Romance*, vol. 1 (London: Macmillan, 1888), p. 173.

16. Aaron, pp. 169–71 (the quotations are from Grave).

17. Aaron, p. 170.

18. Grave, p. 185.

19. H. Gustav Klaus, “The Strike Novel in the 1890s”, *The Rise of Socialist Fiction 1880*–*1914*, ed. Klaus (Brighton: Harvester, 1987), pp. 73–98 (94).

20. E. A. [Amy] Dillwyn, *Chloe Arguelle*, vol. 1 (London: Tinsley Bros., 1881), p. 244.

21.
*Hereford Times*, 18 Jan. 1879; qtd. in David Jones, “The Second Rebecca Riots: A Study of Poaching on the Upper Wye”, *Llafur*, 2.1 (1976): 32–56 (34).

22. The Dau Wynne [Mallt Williams], *A Maid of Cymru: A Patriotic Romance* (London and Carmarthen: Simpkin Marshall and W. Spurrell, [1901]), p. 173. Subsequent references are to this edition and given parenthetically in the text.

23. Katie Gramich discusses the colonial comparisons further in *Twentieth-Century Women’s Writing in Wales: Land, Gender, Belonging* (Cardiff: U of Wales P, 2007), pp. 17–21.

24. Irene Saunderson, *A Welsh Heroine: A Romance of Colliery Life* (London: Lynwood and Co., 1910), p. 81. Subsequent references are to this edition and given parenthetically in the text.

25. Klaus, 80.

26. M. Wynn Thomas, *In the Shadow of the Pulpit: Literature and Nonconformist Wales* (Cardiff: U of Wales P, 2010), p. 27.

27. Thomas, p. 39.

28. “Cymru dros byth” is intended to mean “Wales for ever”. While the grammatically correct form is “Cymru am byth”, this form of the motto was often given in nineteenth-century English publications.

29.
*A Welsh Heroine* is dedicated to the principal of the Calvinistic Methodist theological college in Bala, north Wales, and a marked, but not dogmatic, Christian ideology underpins the novel.

30. Thomas, p. 14.

31. Philip Jenkins, *A History of Modern Wales 1536*–*1990* (Abingdon: Routledge, 2014), p. 196.

32. Allen Raine, *A Welsh Witch: A Romance of Rough Places* (Dinas Powys: Honno, 2013), p. 305.

33. Beale, p. 134.

34. Knight, p. 22.

35. Jane Aaron, introduction, Raine, pp. ix–xxii (xviii).

36. The novelist John Saunders even footnotes his non-fictional source—Louis Simonin’s *Underground Life: Mines and Miners* (1869), a translation from the French—in *Israel Mort, Overman: A Story of the Mine*, vol. 3 (London: Henry S. King, 1876), p. 71.

37. See the interdisciplinary Wellcome Trust-funded project “Disability and Industrial Society: A Comparative Cultural History of British Coalfields 1780–1948”, <http://www.dis-ind-soc.org.uk>.

38. David T. Mitchell and Sharon L. Snyder, *Narrative Prosthesis: Disability and the Dependencies of Discourse* (Ann Arbour: U of Michigan P, 2000), p. 26.

39. Raine, p. 249.

40. Mieneke Te Hennepe, “‘To Preserve the Skin in Health’: Drainage, Bodily Control and the Visual Definition of Healthy Skin 1835–1900”, *Medical History*, 58.3 (2014): 397–421.

41. Arthur McIvor and Ronald Johnston, *Miners’ Lung: A History of Dust Disease in British Coal Mining* (Aldershot: Ashgate, 2013), p. 66.

42. David M. Turner, “Introduction: Approaching Anomalous Bodies”, *Social Histories of Disability and Deformity*, ed. David M. Turner and Kevin Stagg (Abingdon: Routledge, 2006), pp. 1–16.

43. Bradley Lewis, “A Mad Fight: Psychiatry and Disability Activism”, *The Disability Studies Reader*, ed. Lennard J. Davis, 4th ed. (Abingdon: Routledge, 2013), pp. 115–31 (116).

44. Mitchell and Snyder, *Narrative Prosthesis*.

45. We are borrowing Stephen Knight’s terminology here (15). Saunderson’s ethnography is particularly focused on the detail of women’s lives, noting, for instance, the practical clothing worn by a breastfeeding woman: “the front of whose untidy bodice was open, revealing a strip of pink calico, an article of dress greatly affected by the married section of the feminine community of Cwmglo when suckling their infants, being considered by them more ‘handy’ than the opening of hooks and eyes for the purpose” (67). Elsewhere, a curious mixture of sentimental and medical language is used to describe breastfeeding mothers, whose happy tears on finding their husbands saved “mingl[e] [ … ] with the lacteal refreshment wherewith they sought to soothe their little, wailing human burdens” (322).

46. Amy Dillwyn, *A Burglary, or Unconscious Influence* (Dinas Powys: Honno, 2009), p. 82.

47. Dillwyn, *Burglary*, p. 96.

48. E. A. [Amy] Dillwyn, “Nant Olchfa: Chapter XX”, *The Red Dragon: The National Magazine of Wales*, 11 (Jan.–June 1887): 289–99 (296–97). 

